# Inositol Hexakisphosphate-Induced Autoprocessing of Large Bacterial Protein Toxins

**DOI:** 10.1371/journal.ppat.1000942

**Published:** 2010-07-08

**Authors:** Martina Egerer, Karla J. F. Satchell

**Affiliations:** Feinberg School of Medicine, Northwestern University, Chicago, Illinois, United States of America; The Fox Chase Cancer Center, United States of America

## Abstract

Large bacterial protein toxins autotranslocate functional effector domains to the eukaryotic cell cytosol, resulting in alterations to cellular functions that ultimately benefit the infecting pathogen. Among these toxins, the clostridial glucosylating toxins (CGTs) produced by Gram-positive bacteria and the multifunctional-autoprocessing RTX (MARTX) toxins of Gram-negative bacteria have distinct mechanisms for effector translocation, but a shared mechanism of post-translocation autoprocessing that releases these functional domains from the large holotoxins. These toxins carry an embedded cysteine protease domain (CPD) that is activated for autoprocessing by binding inositol hexakisphosphate (InsP_6_), a molecule found exclusively in eukaryotic cells. Thus, InsP_6_-induced autoprocessing represents a unique mechanism for toxin effector delivery specifically within the target cell. This review summarizes recent studies of the structural and molecular events for activation of autoprocessing for both CGT and MARTX toxins, demonstrating both similar and potentially distinct aspects of autoprocessing among the toxins that utilize this method of activation and effector delivery.

## Introduction

Pathogenic bacteria frequently export protein toxins that target eukaryotic intracellular proteins to alter host cell function to the benefit of the infectious pathogen. Different exported toxins employ distinct strategies for translocation of their cytopathic effectors from the bacterium into the host cell. These strategies include direct injection, such as occurs using Type III, Type IV [1], and likely also Type VI secretion [2]. By contrast, some toxins are secreted or released from the bacteria and then bind to host cell surface receptors via a binding (B) component. The B component itself or a separate translocation component then transfers the catalytic subunit or domain across the plasma or endosomal membrane into the cytosol. In some toxins, the B component is a protein subunit assembled with the effector (A) subunit within the bacteria before export (such as cholera toxin [3]), while for other toxins, the B and A subunits are exported separately and then assembled at the surface of the target cell (such as anthrax toxin [4]). Still other toxins are expressed as a single polypeptide that is nicked to separate the A and B domains by endogenous bacterial proteases (such as botulinum toxin [5]) or by host cell proteases during translocation (such as diphtheria toxin [6]). All of these processes succeed in delivering the smaller active effector domains or subunits into the host cell, where they can then access their intracellular protein targets.

Yet, questions have remained as to how single polypeptide toxins that range in size from 250 to 600 kDa deliver their effector domains to the eukaryotic cytosol. A shared strategy for activation of autocatalytic processing upon binding of the eukaryotic signal molecule inositol hexakisphosphate (InsP_6_) has recently been characterized for these toxins. This process represents a novel strategy for toxin activation and subsequent delivery of effectors to target cells.

## Overview of Clostridial Glucosylating Toxins

Clostridial glucosylating toxins (CGTs), also known as large clostridial cytotoxins, are structurally and functionally related toxins produced by different *Clostridium* sp. that range in size from 250 to 308 kDa and have sequence identity from 26% to 76% [7], [8]. *Clostridium difficile* Toxin A (TcdA) and Toxin B (TcdB) are the major virulence factors of clinically important antibiotic-associated diarrheal infections and pseudomembranous colitis [9]. Recent studies revealed that, while some *C. difficile* strains produce both toxins, only TcdB is essential for virulence [10]. Other significant members of the CGT family are Lethal Toxin from *C. sordellii* (TcsL) and the α-toxin from *C. novyi* (Tcnα). These clostridia are more rare causes of disease, but have been associated with particularly severe invasive infections, including gas gangrene and toxic shock following abortions or gynecological procedures [11]–[14].

**Figure 1:**
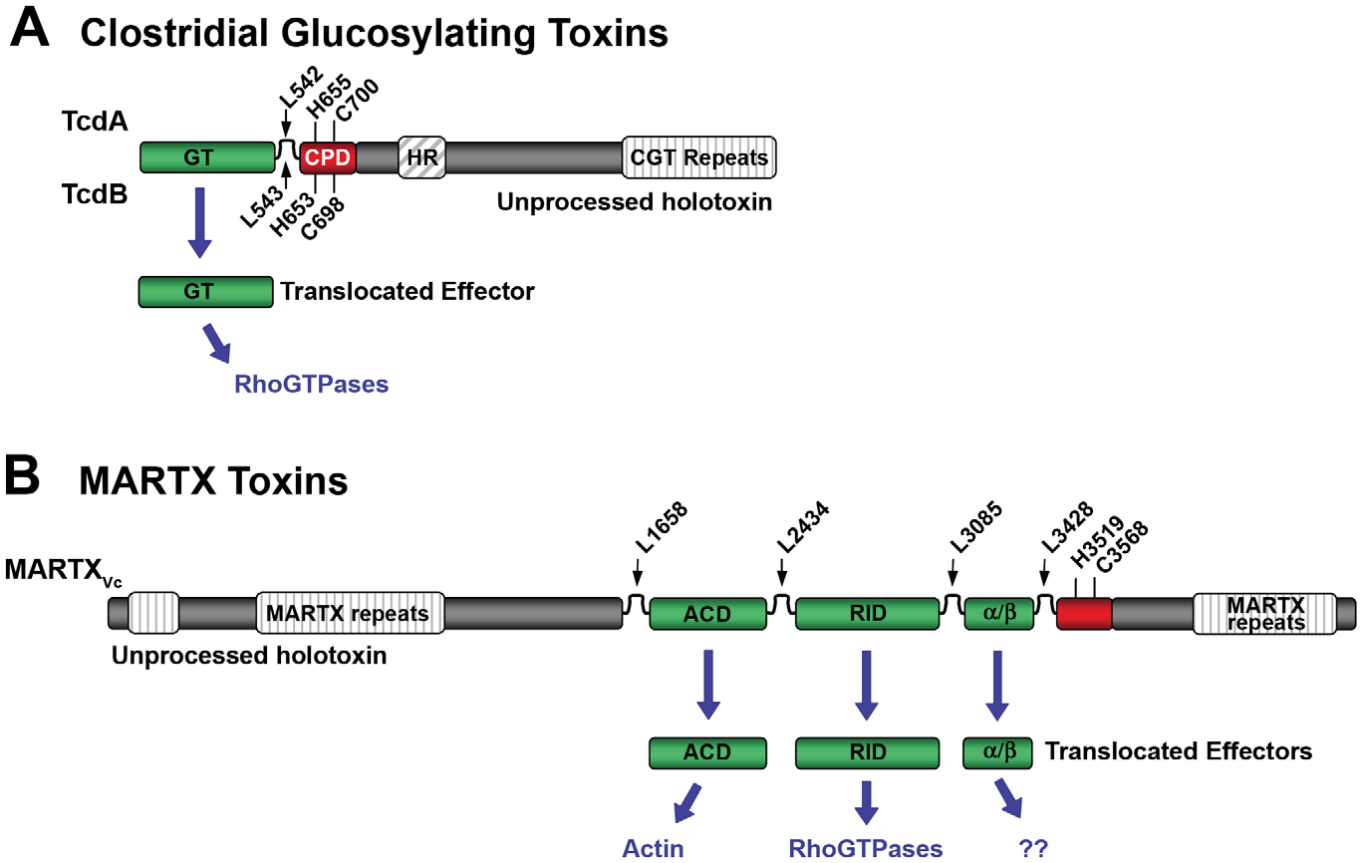
Schematic diagrams representing CPD-dependent autoprocessing sites within CGTs and MARTX toxins. Diagrams are shown for (A) CGTs represented by TcdA and TcdB or (B) MARTX toxins represented by MARTX_Vc_. In (A), the CGT holotoxins contain C-terminal repeats required for receptor interactions and a hydrophobic region (HR) postulated to function in translocation of the GT across the membrane of the endosome. Upon autoprocessing, the catalytically active glucosyltransferase effector (GT) is delivered to cells where it targets RhoGTPases. In (B), the MARTX holotoxin contains both N- and C-terminal repeats that likely function in translocation. Upon autoprocessing, MARTX_Vc_ delivers three effectors that have distinct cellular targets as indicated. For both diagrams, the CPD catalytic Cys and His are marked, as are processing site Leu residues (see Table 1) found in unstructured segments between effectors (indicated by arrows). For CGTs, sequence numbering above the diagram represents TcdA while numbering below the diagram represents TcdB. For MARTX_Vc_, sequence numbering is based on the original annotation of the *rtxA* gene by Lin et al. [37] and may be different than that found in cited references.

The CGTs are organized in a multidomain structure [15], including a biologically active effector domain, a middle translocation domain, and a C-terminal receptor-binding domain [8] (Figure 1A). To enter eukaryotic target cells, the secreted CGTs bind to extracellular receptors and follow the “short trip model” of exotoxin uptake [16]. After receptor-mediated endocytosis, a vesicular H^+^-ATPase leads to acidification of the early endosomes, inducing a conformational change and an increase in hydrophobicity [17]. A small hydrophobic region of the protein is proposed to form a pore through which the N-terminus-localized glucosyltransferase (GT) domain is translocated into the cytosol [18]–[20]. Using UDP-glucose (UDP-*N*-acetylglucosamine for Tcnα) as a co-substrate, the GT monoglucosylates Rho family GTPases. The covalent modification occurs at a specific threonine residue found within the GTP binding pocket (Thr37 in Rho, Thr35 in Rac), thereby preventing activation of the small GTPases by exchange of GTP for GDP. Ultimately, the accumulation of inactive GTPases results in reorganization of the cytoskeleton and other morphological changes [21].

## Overview of Multifunctional-Autoprocessing RTX Toxins

Multifunctional-autoprocessing RTX (MARTX) toxins are larger toxins that range in size from 350 to 600 kDa [22]. The MARTX toxin of *Vibrio cholerae* (MARTX_Vc_) has been linked to virulence, in which the toxin functions during early infection to promote colonization, possibly by inactivating cellular innate immunity [23]–[25]. The MARTX toxins from both human [26]–[29] and aquatic animal [30] infectious *Vibrio vulnificus* strains (MARTX_Vv_) and the fish pathogen *Vibrio anguillarum* (MARTX_Va_) [31] have likewise been associated with virulence. In addition, putative MARTX toxins have been identified in at least 13 other sequenced Gram-negative bacteria, including *Proteus*, *Aeromonas*, *Yersinia*, and *Photorhabdus* spp. [22], [32]–[36], suggesting that additional pathogens require these toxins as virulence factors.

Similar to CGTs, the MARTX toxins are modular in structure, but are typified by the presence of extensive repeats at both the N- and C-termini [22], [37]. These repeats are postulated to form the translocation structure for transfer of centrally located effector domains to the cytosol [22] (Figure 1B). For MARTX_Vc_, cytopathic effects occur in the presence of inhibitors of endocytosis [38]–[40], suggesting that endocytosis is not required for MARTX toxin entry, as it is for the CGTs.

Among the various MARTX toxins, a total of ten potential effectors have been identified, although each independent toxin has an assortment of only one to five [22]. The best-characterized MARTX effector is the actin crosslinking domain (ACD), which introduces a Glu270-Lys50 isopeptide linkage between actin monomers by a mechanism similar to that for glutamine synthetases [41], [42]. Another MARTX effector inactivates RhoGTPases by an unknown mechanism [43], although a recent bioinformatics study suggested this domain is a thiol protease [44]. The remaining eight potential effectors are domains of unknown function, even though two share sequence homology with *Photorhabdus luminescens* and *Pasteurella multocida* toxins and one is conserved with the alpha/beta hydrolase family of proteins [22].

## Both CGT and MARTX Toxins Undergo Autoprocessing by a Conserved Cysteine Protease Domain

Early studies of the CGTs postulated that they would undergo enzymatic processing after exposure to low pH [17], [45]. Subsequent in vivo studies demonstrated that only the 60-kDa GT effector of TcdB is delivered to the cytosol, while a larger C-terminal fragment remains in the membrane fraction [20]. This processing of TcdB also occurred in vitro after residue Leu543, with a strict dependence upon addition of eukaryotic cell lysate [46] (Figure 1A). These studies of TcdB were initially interpreted as indicating processing by a host cell–encoded protease, similar to the mechanism for maturation of diphtheria toxin and other bacteria toxins [6]. However, protein-free extracts also stimulated TcdB processing, indicating autocataytic cleavage [47].

Similarly, early studies of MARTX_Vc_ postulated that the ACD would need to be released by proteolysis to access the entire actin pool [40]. In fact, it has been demonstrated that MARTX_Vc_ is autoprocessed at four positions located before and after its three effector domains, resulting in the release of these domains from the holotoxin [48], [49] (Figure 1B).

The autoprotease domain responsible for MARTX_Vc_ processing was recognized first as a 25-kDa domain within MARTX_Vc_ that affected cell viability when ectopically expressed in eukaryotic cells [50]. This cytotoxicity was disrupted by mutation of a single Cys or His residue, and analysis of protein expression patterns revealed that the mutant proteins were the predicted size, while the wild-type protein was cleaved of its N-terminus. Studies with recombinant protein confirmed autoprocessing after Leu3428 and, similar to TcdB, processing was strictly dependent upon addition of protein-free cell cytosol extracts [50]. Mutation of the critical Cys in the full-length toxin significantly reduced the ability of the toxin to induce actin crosslinking, confirming autoproteolysis due to this cysteine protease domain (CPD) enhanced toxin function [50].

After its discovery within MARTX_Vc_, the CPD was found to be conserved within all MARTX toxins and also in all CGTs, with common alignment of the processing sites and the catalytic Cys and His residues [50], [51]. Furthermore, for TcdB, mutation of the analogous Cys and His residues reduced cytotoxicity of the full-length toxin, and disrupted processing of recombinant CPD protein as well [51], [52]. Thus, it was recognized that both the CGTs and MARTX toxins share a common mechanism for autocatalytic processing inducible by protein-free eukaryotic cell cytosol and that autoprocessing is essential for optimal cytotoxicity.

## InsP_6_: The Inducer of Autoprocessing

To identify the molecule in cell cytosol required to induce CPD for autoprocessing, cell extracts that stimulated processing of TcdB were fractionated and analysis of active fractions by mass spectrometry supplied spectra with similarities to inositol phosphates [47]. Incubation of TcdB with several inositol phosphates indicated that InsP_6_ induced the most efficient autoprocessing activity [47]. Similar studies indicated that InsP_6_ is likewise the most effective stimulator of autoprocessing of the MARTX_Vc_ CPD [53].

As a signal molecule for the eukaryotic intracellular environment, InsP_6_ (also known as phytic acid) is an excellent selection for bacterial toxins, since the molecule is ubiquitous in eukaryotes but absent in bacteria. Furthermore, InsP_6_ is the most abundant of the inositol phosphates, is maintained within cells at relatively constant levels of 10–60 µM, and is generally freely soluble in the cytoplasm [54]. Within mammalian cells, InsP_6_ may function as a high concentration storage molecule for phosphate as it does in plant seeds or as a highly charged buffer for cation- or protein-dependent processes. More recent studies have linked InsP_6_ to numerous cellular processes, including vesicle recycling, mRNA transport out of the nucleus, and as a co-factor for a DNA-dependent protein kinase [54]. Regardless of its normal function, InsP_6_ is a molecule constantly present in high concentrations in the eukaryotic cytosol, assuring that induction of CGT and MARTX CPDs occurs only after completion of translocation of effector domains to the cytosol, regardless of whether translocation requires endocytosis or transfer directly across the plasma membrane.

## InsP_6_ Binding to the CPDs

**Figure 2:**
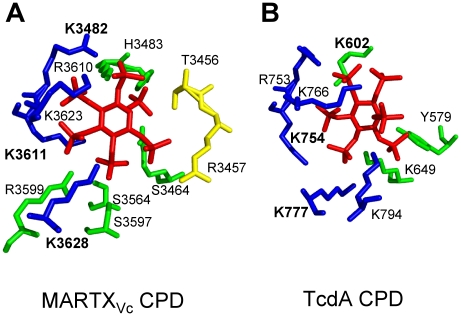
Side chain residues from CPD that contact InsP_6_ in the structural models derived from crystal structures of MARTX_Vc_ and TcdA CPD. All key residues that contact InsP_6_ in the CPD of (A) MARTX_Vc_ and (B) TcdA are shown labeled with a single letter code, with the three Lys residues determined to be most critical for InsP_6_ binding shown in bold text. Interestingly, despite strong conservation of the critical Lys residues in the primary amino acid sequence, contacts with InsP_6_ and the orientation of InsP_6_ differ in the two structures. Diagram is colored to represent residues originating from the N-terminal strand (yellow), the core structure (green), and β-strands G_1_-G_5_ (blue), a structure also known as β8-β12 or the β-flap. Structural models were based on PDB (A) 3FZY [48] and (B) 3HO6 [57], and figures were prepared with MacPyMol software (DeLano Scientific).

While both CGT and MARTX CPDs are induced for autoprocessing by InsP_6_, there are differences in these proteins revealed by crystallography and InsP_6_ binding studies that suggest slightly different mechanisms of activation. Mutational studies [53], [55]–[57] and analysis of four independent crystal structures [48], [49], [56], [57] revealed that binding of InsP_6_ to the CPD involves contact of the six negatively charged phosphate groups within a positively charged pocket of the CPD (Figure 2). The most significant binding contacts of MARTX_Vc_ with InsP_6_ involve Lys3482, Lys3611, and Lys3623. Other Lys, Arg, and positively charged residues that form the binding pocket are not essential for binding, but do contribute to the high affinity of the MARTX_Vc_ CPD for InsP_6_ [53], [56]. In TcdB, Lys600 (analogous to Lys3481 of MARTX_Vc_) is likewise essential for binding of InsP_6_ [55], while other conserved Lys and Arg residues also contact InsP_6_ [55], [57]. Interestingly, overlay of the structures of the CPD from MARTX_Vc_ and TcdA CPDs revealed that the orientation of InsP_6_ in the binding pocket is not conserved [57] (Figure 2), which is a surprise since amino acid sequence alignments show strong conservation of the Lys and Arg residues that form the binding pocket [55], [57]. However, this difference in the structure of the binding pocket may in part account for variances revealed in studies of InsP_6_ binding and CPD activation for the different CPDs.

Intramolecular processing of purified MARTX_Vc_ CPD was found to be optimal in the range of 0.001–1 µM InsP_6_ [53], [56], and binding of InsP_6_ occurred with affinities ranging from 0.2 to 1.3 µM InsP_6_ [48], [53], [56]. The ability of MARTX_Vc_ CPD to complete autoprocessing in vitro at concentrations below the dissociation constant reflects the recycling of InsP_6_ released from processed CPD back to predominantly unprocessed protein [53], since processed MARTX_Vc_ CPD has a 500-fold reduced affinity for InsP_6_ [48].

By contrast, activation of purified TcdB CPD autoprocessing requires 2 µM InsP_6_ [51], a concentration near the determined K_d_ of 2.3 µM [55]. Furthermore, recombinant TcdB CPD that mimics protein processed after Leu543 binds InsP_6_ with a similar affinity as full-length TcdB and unprocessed recombinant CPD [55], suggesting that TcdB CPD, unlike MARTX_Vc_ CPD, does not have an altered affinity for InsP_6_ after processing.

Although the dissociation constant has not as yet been determined for TcdA, processing studies indicate that its ability to bind InsP_6_ may be less efficient than TcdB. Whereas full-length TcdB is cleaved to completion at InsP_6_ concentrations of 2–10 µM [47], [51], full-length TcdA does not autoprocess at 10 µM InsP_6_ and concentrations up to 10 mM have been used for experimentation [47], [51]. However, recombinant TcdA CPD does autoprocess at concentrations as low as 5 µM [57], suggesting that the affinity of TcdA CPD for InsP_6_ could be near to that of TcdB, but not accurately reflected in holotoxin cleavage assays.

Despite the apparent differences in the structure of the InsP_6_ binding pocket and binding kinetics, the high affinity of the unprocessed CPDs for InsP_6_ indicates that autoprocessing of MARTX_Vc_, TcdB, and TcdA would all proceed efficiently at InsP_6_ concentrations of 10–60 µM that are found in the eukaryotic cell cytosol [54]. Thus, all of the studied CGT and MARTX toxins would be autoprocessed and effectively deliver their effector domains within the in vivo environment.

## Structural Arrangement of the CPD Catalytic Site

**Figure 3:**
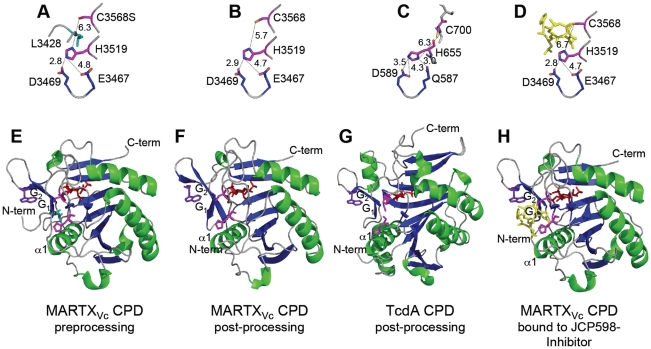
Crystal structures of MARTX_Vc_ and TcdA CPDs. Crystal structures of the (A–D) CPD catalytic sites with distances between residues designated in angstroms and (E–G) the CPD proteins are shown at various stages of processing. (A, E) Pre-processing and (B, F) post-processing structures of MARTX_Vc_ CPD bound to InsP_6_ (PDB 3FZY [48] and PDB 3EEB [56], respectively). (C, G) Post-processing structures of TcdA CPD bound to InsP_6_ (PDB 3HO6 [57]). (D, H) Post-processing structure of MARTX_Vc_ bound to *z*-Leu-Leu-azaLeu-epoxide inhibitor JCP598 as a surrogate substrate representing the structure of CPD after reactivation (PDB 3GCD [49]). Structures are identically oriented at a conserved Trp (purple) in the G_1_/G_2_ β-hairpin that is critical to InsP_6_ induction of autoprocessing [56]. The catalytic Cys and His residues are shown in pink with InsP_6_ present at the backside of each structure in red. The P1 Leu (turquoise) is found only in the unprocessed structure (A) with the scissile bond oriented between the catalytic residues. Figures were prepared with MacPyMol software (DeLano Scientific).

The CPD catalytic dyad is composed of one His and one Cys residue separated by ∼6 Å in both MARTX_Vc_ and TcdA structures [48], [56], [57] (Figure 3A–3D). The distance between the catalytic residues indicates that the Cys is not activated by protonation from His, but rather suggests that the Cys is substrate-activated by close alignment of the scissile bond, while the His functions solely to protonate the leaving group [48], [57].

In addition, Asp and Glu residues play an essential function in proteolysis. Mutation of TcdB Asp567 or TcdA Asp589 disrupted autoprocessing [51], [57] and eliminated cytopathic effects when added to HeLa cells [55]. By contrast, mutation of the analogous Asp3469 in the MARTX_Vc_ CPD did not affect autoprocessing [53]. Analysis of the structural models indicates that this conserved Asp residue functions in both proteins to properly orient the catalytic His residue [48], [57] (Figure 3A–3D). However, in the MARTX_Vc_ CPD structure, this function is shared with residue Glu3467, such that only a double Asp/Glu mutant is defective for function [53].

The closest known cysteine proteases that share this structural arrangement of the catalytic site are caspase-1 and gingipain R [58], [59]. Similar to these other proteases [60], [61], the CPDs are resistant to cysteine protease inhibitor E64 [50], but sensitive to *N*-ethylmaleimide, iodoacetamide, or chloromethyl ketones [48], [50], [51]. Thus, the CGT and MARTX toxin CPDs are grouped together with caspase-1 and gingipain R in the CD clan of cysteine proteases, but form a new family, the C80 family (http://merops.sanger.ac.uk, [62]). The CPD proteases have also been incorporated into a larger CPD_adh_ family of putative bacterial and eukaryotic peptidase that are proposed to share a similar fold in the catalytic site [63].

## Structure-Based Modeling of InsP_6_-Induced Activation of the CPDs

As the binding site for InsP_6_ occurs on the opposite side of the protein from the catalytic site (Figure 3E–3H), it was recognized that there must be a mechanism to transduce the binding signal across the entire protein structure [56]. Translocation of effector domains of both CGTs and MARTX toxins is predicted to involve transit through a pore for entry into the cytosol, and thus the CPD is likely partially unfolded when it is first presented to the InsP_6_-rich environment of the cytosol [15], [22]. Consistent with this model, apo-CPD in the absence of InsP_6_ for both MARTX_Vc_ and TcdA is highly sensitive to proteolysis [48], [57]. Nuclear magnetic resonance (NMR) studies of the TcdA CPD indicated that the apo-protein is folded, but undergoes a significant conformational reorganization upon InsP_6_ binding [57]. This finding is consistent with an observed high negative enthalpy and entropy and a 14°C thermal stability shift upon binding of InsP_6_ to MARTX_Vc_ CPD, suggesting that this protein undergoes a major structural rearrangement that also stabilizes the protein structure [48].

**Figure 4:**
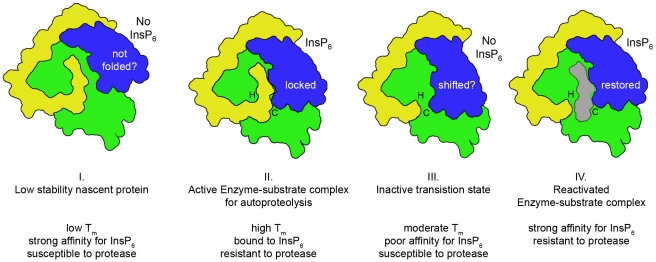
Proposed model for cooperative activation and reactivation of MARTX_Vc_ CPD by InsP_6_. I. Apo-CPD without InsP_6_ is an unstable protein susceptible to thermal denaturation at physiological temperature. The core structure (green) is folded but the β-flap (blue) is susceptible to proteolysis, indicating it may be only partially structured. II. Upon binding InsP_6_, the structure rearranges such that the N-terminus (yellow) becomes locked within the active site between the catalytic Cys (C) and His (H) in a rigid alignment amenable to substrate-activated autoprocessing. III. After autoprocessing, the MARTX_Vc_ CPD enters a transitional state that has distinct biochemical properties, including a 500-fold reduced affinity for InsP_6_. IV. After first binding a new substrate (grey) and then a new molecule of InsP_6_, the enzyme–substrate complex structure of the MARTX_Vc_ CPD is restored for additional processing events. Figure is based on multisite processing model for MARTX_Vc_ proposed by Prochazkova et al. [48]. Current evidence from NMR studies supports the idea that stage I and II also occur for TcdA [57]. However, binding studies with TcdB suggest CGTs likely do not undergo stage III deactivation or stage IV reactivation [55].

Upon binding InsP_6_, the structure of the MARTX_Vc_ CPD adopts the stable conformation amenable to X-ray crystallography (Figure 3E–3G). The CPD is composed of a seven-stranded β-sheet with two α-helices flanking the sheet and one capping the sheet [56]. An additional five β-strands at the C-terminus form a subdomain, known as the β-flap, that is loosely attached to the core of the protease [56]. By contrast, the TcdA CPD is larger and consists of a nine-stranded β-sheet flanked by five α-helices (Figure 3G) [57]. In both proteins, the N-terminus is an unstructured strand wrapped around the outside of the protein and attached to the core structure by embedding of large hydrophobic residues [48], [57]. At the extreme N-terminus of the CPD, the P1 Leu residue found immediately before the scissile bond is buried in a hydrophobic pocket (Figure 3E) [48]. Mutagenesis studies and structural analysis [48], [49] have demonstrated that Leu is the only residue that can be accommodated at this position. On either side of the Leu, any residue can occur but there is a preference for small residues, creating a consensus sequence of small-Leu-small ([48], [49] and Table 1). The G_1_ β-strand (also known as β8) forms part of the hydrophobic pocket [56], and conserved Leu and Val residues on this strand make direct contact with the P1 Leu before the scissile bond [48]. This G_1_ strand is antiparallel to the G_2_ β-strand (also known as β9), a strand that contributes positively charged amino acids that make contact with InsP_6_ [56]. The current model for activation of the MARTX_Vc_ CPD proposes that binding of InsP_6_ alters the structure of this antiparallel β-hairpin, resulting in stabilization of the N-terminus within the hydrophobic pocket [48] and possibly reorientation of the catalytic Cys [56]. The net effect is to orient the scissile bond between the catalytic Cys and His residues, resulting in substrate-activated autoprocessing [48] (Figure 4). A similar mechanism has been proposed for the activation of TcdA CPD [57].

**Table 1:** InsP_6_-induced autoprocessing CGTs and MARTX toxins.

Toxin Group	Bacterial Toxin (Abbreviation)	Number of Effectors^a^	Cellular Targets^a^	Processing Sites^b^	Reference
**CGT**	*C. difficile* Toxin A (TcdA)	1	RhoGTPases	GGSL_542_↓SED (p)	[47], [51]
	*C. difficile* 8864 Toxin B (TcdB_8864_)	1	RhoGTPases	EGAL_543_↓GED (m)	[46]
	*C. difficile* 10463 Toxin B (TcdB)	1	RhoGTPases	EGSL_543_↓GED (m)	[46]
	*C. novyi* Alpha toxin (Tcnα)	1	RhoGTPases	GRTL_548_↓NYE (p)	[46], [47]
	*C. sordellii* Lethal toxin (TcsL)	1	RhoGTPases	EGAL_543_↓GED (p)	[46]
**MARTX**	*V. cholerae* MARTX_Vc_	3	Actin, RhoGTPases, ??	LESL_1658_↓SAV (m)	[48]
				LHAL_2434_↓GET (m)	[48], [49]
				LDAL_3085_↓SGN (m)	[48], [49]
				KEAL_3428_↓ADG (m)	[50]
				QQGL_3402_↓DTT (a)	[49]
				NDHL_3419_↓AVV (a)	[48]
	*V. vulnificus* MARTX_Vv_	5	RhoGTPases, ??	KGSL_4089_↓SGA (m)	[49]
	*P. luminescens* MARTX_plu1341_	1	??	LQAL_2538_↓SGK (p)	[49]
	*P. luminescens* MARTX_plu1344_	2	??	SGAL_2962_↓MSQ (p)	[49]
	*P. luminescens* MARTX_plu3217_	1	??	LDWL_2408_↓SGK (m)	[49]
				VEAL_2405_↓DWL (a)	[49]
	*P. luminescens* MARTX_plu3324_	1	??	LEGL_2418_↓SGT (p)	[49]

a Based on analysis of effector domains as reviewed in [15], [22].

b Processing site is indicated by inverted arrow. m, processing site as mapped experimentally by N-terminal sequencing or mass spectrometry; p, processing site predicted by homology to mapped processing site from closely related toxin; a, alternative processing site identified by mass spectrometry. Numbering of MARTX_Vc_ processing sites is based on amino acid sequence as originally annotated in [37] and may be different than that found in cited references. For other MARTX toxins, not all processing sites are known and only those previously reported in the literature are listed.

## Multisite Processing of MARTX_Vc_

The CGT toxins require only a single processing event to release their GT effector domain (Figure 1A), and this may account for why activation apparently requires a structural change between two apparently stable conformations at approximately 5 µM InsP_6_ [57]. By contrast, MARTX toxins must undergo processing at multiple sites to release each of the effectors independently [48], [49] (Figure 1B). A simplistic model of multisite processing would predict that activation of the CPD by binding of InsP_6_ results in transition to a constitutive “on” conformation after which it processes all accessible sites immediately [56]. Yet, biochemical studies described above indicated that after processing of its own N-terminus, MARTX_Vc_ CPD converts to an inactive conformation with a 500-fold reduced affinity for InsP_6_ (K_d_ = 100 µM) [48] (Figure 4). Since this concentration is above the upper limit of the in vivo concentration of InsP_6_ [54], only a small fraction of processed CPD would bind InsP_6_ in vivo, limiting the likelihood of multisite processing. However, it was found that reactivation of MARTX_Vc_ CPD for high affinity binding of InsP_6_ occurs after insertion of a new substrate into the hydrophobic pocket, indicating cooperativity of substrate and InsP_6_ binding [48]. Both binding studies [48] and crystallography [49] (Figure 3G) have shown that chloromethyl ketone and epoxide inhibitors bound to Leu can substitute for a new substrate to restore the protein to an active enzyme-substrate complex. Upon reactivation, the protein is able to process any other available processing sites [48], [49], although there is a preference for processing within the same molecule of MARTX_Vc_, indicating there may be a physical association of the CPD with the effector domains [48].

## Potential of CPD as a Target for Therapeutic Intervention

The discovery of InsP_6_-induced autoproteolysis as a critical stage for activation and effector delivery for large bacterial toxins raises the potential for anti-toxin small molecules to be developed as therapeutics. TcdB is the most significant virulence factor of *C. difficile* [9], [10], and it is conceivable that specific TcdB anti-toxin drugs could be combined with antibiotic and anti-toxin antibody therapies for treatment of recurrent antibiotic-associated diarrhea [64]. In addition, the contribution of MARTX_Vv_ to *V. vulnificus* septicemic infection is significant [26]–[29], suggesting that anti-MARTX_Vv_ CPD therapeutics may be of interest, particularly since there are currently no anti-toxin treatments for these rapidly progressing fatal infections. By contrast, clinical intervention against any domain of MARTX_Vc_ during cholera disease is impractical since animal studies suggest that MARTX_Vc_ functions only during the earliest stage of infection, prior to the onset of symptoms [24], [25]. Indeed, classical *V. cholerae* strains responsible for severe cholera during the fifth and sixth pandemics have a natural deletion in the *rtxA* gene that encodes MARTX_Vc_, demonstrating that it is dispensable for late stage infection [37].

For research purposes, potent small molecule inhibitors of the MARTX_Vc_ CPD activity have been identified. These include peptidyl (acyloxy)methyl ketone epoxide [49] and chloromethyl ketone [48] inhibitors in which the amino acid leucine is linked to the functional group independently or as part of a tripeptide. Both classes of inhibitor are cysteine reactive and become covalently linked to the catalytic cysteine (Figure 3D). However, analysis of the pre-processed form of MARTX_Vc_ CPD revealed the N-terminus is bound within the active site prior to InsP_6_ binding, which occludes access of the catalytic Cys to protease inhibitors. Thus, inactivation of the CPD with Cys reactive inhibitors requires long incubation times of up to 30 minutes [48]. Yet, upon initial intramolecular processing immediately upstream of the CPD, the catalytic Cys is exposed, facilitating rapid inhibition of subsequent processing events that release effectors [48], [49]. Consistent with these in vitro findings, exogenous addition of the membrane permeant *z*-Leu-Leu-azaLeu-epoxide inhibitor JCP598 to culture cells reduced actin crosslinking in vivo [49], suggesting inhibitors could be useful at a critical point after CPD translocation.

Similar inhibition studies using the more clinically relevant TcdB remain to be performed. Since the CGTs are only processed one time (Figure 1A), there is a concern that cysteine reactive inhibitors would be ineffective if the N-terminus bound in the active site blocks access to the catalytic Cys. A structure of the enzyme-substrate complex of TcdA or TcdB CPD is not yet available and inhibition by *N*-ethylmaleimide has been performed only with 30 minutes of incubation [51]. Thus, it is unknown if the accessibility of the Cys will be blocked similar to MARTX_Vc_ CPD. As described above, binding of InsP_6_ to the recombinant TcdB CPD protein with the P1 leucine removed has been measured and shown to be similar to that with the Leu attached [55]. These results thereby suggest the association of the N-terminus with the TcdB catalytic site and relevant exposure of the catalytic site to inhibitors may differ from MARTX_Vc_ CPD. Hence, the potential for inhibition of InsP_6_-induced autoprocessing by CPDs as a therapeutic intervention against TcdB merits further exploration. If access to the cysteine is indeed found to be blocked, the CPD could still be a suitable target for therapeutics, but with molecules that mimic InsP_6_ itself to promote processing outside of cells, potentially disrupting the entire translocation/activation process.
